# Aspartate aminotransferase to platelet ratio can reduce the need for transient elastography in Chinese patients with chronic hepatitis B

**DOI:** 10.1097/MD.0000000000018038

**Published:** 2019-12-10

**Authors:** Wei Yue, Yan Li, Jiawei Geng, Ping Wang, Li Zhang

**Affiliations:** a Faculty of Environmental Science and Engineering, Kunming University of Science and Technology; b Department of VIP Internal Medicine, The First People's Hospital of Yunnan Province; c Department of Infectious Disease, The First People's Hospital of Yunnan Province, Kunming, Yunnan, China.

**Keywords:** APRI, CHB, China, FIB-4, GPR, non-invasive diagnosis, TE-FibroTouch

## Abstract

In the absence of liver biopsy and transient elastography (TE), aspartate aminotransferase to platelet ratio (APRI), fibrosis-4 score (FIB-4), and gammaglutamyl transpeptidase to platelet ratio (GPR) are simple and inexpensive methods for the detection of liver fibrosis. Aims: We compared the performance of APRI, FIB-4, and GPR scores against TE in predicting the presence of liver fibrosis and cirrhosis, determined the optimal cut-off values for fibrosis and cirrhosis prediction, and reviewed the need for further TE assessment in resource-limited areas in China. Methods: TE and basic laboratory tests were performed in 2014 consecutive patients with chronic hepatitis B (CHB), and then compared to APRI, FIB-4, and GPR. Results: For the detection of significant fibrosis, the areas under the receiver operating characteristic (AUROC) curves for APRI, FIB-4, and GPR were 0.83, 0.75, and 0.77, respectively. For the detection of cirrhosis, the AUROC curves for APRI, FIB-4, and GPR were 0.90, 0.84, and 0.84, respectively. The cutoff of APRI was 0.35, with 78% sensitivity and 63% negative predictive value (NPV), to exclude significant fibrosis (F ≥ 2). At an APRI of 0.6, results showed a 94% specificity, 100% positive predictive value (PPV) and 7.9 positive likelihood ratio (PLR) in detecting significant fibrosis. Thus, patients with an APRI of <0.35 or >0.6 demonstrated correct prediction of liver fibrosis. These results translated to 1250 out of the 2014 patients avoiding the need for TE with a diagnostic accuracy of >80%. Conclusions: The APRI score accurately assessed fibrosis and reduced the need for TE in almost two-thirds of Chinese patients with CHB.

## 1 Introduction

Hepatitis B virus (HBV) infection is of considerable concern in China, with more than 93 million cases currently, of which 20 million exhibit chronic hepatitis B (CHB).^[1]^ Liver cirrhosis and cancer caused by CHB also result in more than 30,000 deaths each year.^[1]^ Thus, assessment of liver fibrosis is an important determinant of both stage and prognosis, as well as therapeutic decision-making and optimal treatment timing in CHB patients.

Liver biopsy has long been considered the gold standard for the diagnosis and prognosis of liver disease; however, this procedure is invasive and expensive, and a lack of trained personnel often restricts its use in low-income areas.^[2]^ TE is a non-invasive assessment tool with rapid acquisition. In China, the FibroTouch liver fibrosis diagnostic TE system has been widely applied since 2013.^[3,4]^ Many studies have shown TE to have excellent agreement with liver biopsy in patients with hepatitis B and C.^[5,6]^ However, while TE demonstrates good diagnostic accuracy in quantifying liver fibrosis and cirrhosis,^[2]^ the cost and accessibility of TE equipment (including FibroScan and FibroTouch) have restricted its application in resource-limited countries. The device is expensive and often only accessible in a limited number of hospitals in developing areas, including Yunnan Province, China.

A systematic review of the cost effectiveness of TE compared with liver biopsy showed that TE is economical but can incur added costs of almost US $3000.^[7]^ More importantly, accessibility of TE can be an issue in resource-limited settings. Therefore, the development of non-invasive tests to screen patients from low-income areas, who would otherwise require further TE or liver biopsy, is necessary.

In 2015, the World Health Organization (WHO) published guidelines for the management of CHB infection in areas with limited or no access to liver biopsy or TE, recommending the use of non-invasive tests to detect significant liver fibrosis and cirrhosis.^[8]^ And Imanieh et al investigated APRI may be used as a simple test to evaluate the liver fibrosis in children with genetic liver diseases.^[9]^ The APRI, FIB-4, and GPR are attractive non-invasive tools, particularly in resource-poor areas, and are reliable predictors of hepatic fibrosis in patients with chronic hepatitis C (CHC).^[10–12]^ In addition, the costs of liver biopsy and TE for each patient are $956.61 and $51.00, respectively, whereas the costs for APRI and FIB-4 are $4.05 and $4.40, respectively, thus representing substantial savings. To date, however, few studies have evaluated their performance in CHB or their performance against TE (rather than liver biopsy), especially in means-restricted countries.

Therefore, we evaluated and compared the performances of APRI, FIB-4 and GPR against TE in predicting the presence of liver fibrosis and cirrhosis and determined the best cut-off scores for predicting the likelihood of fibrosis and cirrhosis and the need for further TE or liver biopsy assessment in resource-limited areas in China (e.g., Yunnan Province). The goal of this study was to minimize the need for TE and liver biopsy, rather than to replace them completely, and thereby reduce the need for invasive procedures as well as the costs incurred by patients, hospitals, and governments.

## 2 Materials and methods

### 2.1 Study setting and participants

Yunnan Province is a low-income area located in southwest China, with an intermediate to high prevalence of CHB. Unfortunately, many local hospitals lack trained personnel to perform liver biopsies and TE equipment is limited. Thus, we aimed to compare non-invasive fibrosis markers with TE results to determine whether some patients can avoid TE testing.

Consecutive patients diagnosed with CHB during liver clinic follow-up and who underwent TE and basic laboratory tests were selected from the First People's Hospital of Yunnan Province between 2015 to 2017. Here CHB was diagnosed by positive serology tests for serum hepatitis B surface antigen (HBsAg) for at least 6 months. Exclusion criteria included chronic liver disease due to other causes or co-infection with HCV, hepatitis D virus, or HIV and alcohol consumption in excess of 20 g/day. Patients with alanine transaminase (ALT) levels more than 3 times the upper limit of normal (ULN, 40 IU/L) were also excluded. No enrolled patients received any treatment. This study was approved by the First People's Hospital of Yunnan Province Ethics Committee.

### 2.2 Laboratory analyses

All patients underwent baseline examination, which included aspartate transaminase (AST), ALT, albumin, albumin/globulin (A/G), total bilirubin (TBIL), direct bilirubin (DBIL), γ-glutamyl-transpeptidase (γ-GT) levels and platelet counts (PLT). Markers of the hepatitis virus, including HBsAg, hepatitis B e-antigen (HBeAg), anti-HBe, and serum HBV-DNA concentration, were also recorded. The APRI, FIB-4, and GPR scores were then calculated using the following formulae:

APRI: (AST [IU/L]/ULN of AST) / platelet count (10^9^/L) × 100^[13]^
FIB-4: (age [years] × AST [IU/L]) / (platelet count [10^9^/L] × (ALT [IU/L])^1/2^)^[14]^
GPR: (GGT[IU/L]/ULN of GGT) / platelet count (10^9^/L) × 100 ^[15]^

### 2.3 Transient elastography

For fibrosis assessment, TE was employed (FibroTouch-B China). The results of the liver elasticity measurements were expressed in kilopascals (kPa) within the range of 2.5 to 75 kPa. Liver stiffness assessment is generally considered reliable when the following criteria are fulfilled: 10 valid measurements, success rate of >60%, and ratio of interquartile range to median (IQR/M) of ≤30%.^[16–18]^ All patients fasted for at least 3 hours prior to examination.^[19]^ The M transducer was used for all TE examinations to avoid potential bias in interpreting the results in kPa.^[20–22]^ All liver stiffness measurements (LSM) were related to the validated liver fibrosis stages with cutoff values of: <7.3 kPa = F0-1; 7.3 kPa ≥ F2; 9.7 kPa ≥ F3; 12.4 kPa = F4.^[23]^

### 2.4 Statistical analysis

Continuous variables were expressed as means ± SD or median (inter-quartile range [IQR]), as appropriate. The different non-invasive markers were compared with FibroTouch TE values. Bivariate Spearman's rank correlation coefficient was used to analyze the correlations among APRI, FIB-4, and GPR scores with TE grades. Their diagnostic accuracies were estimated by calculating the areas under the receiver operating characteristic (AUROC) curves. Diagnostic performances of the APRI scores were analyzed separately according to sensitivity (Se), specificity (Sp), positive likelihood ratio (PLR), negative likelihood ratio (NLR), negative predictive values (NPV), and positive predictive values (PPV). Statistical significance was considered at *P* < .05. Statistical analysis was performed using SPSS 17.0 software.

## 3 Results

### 3.1 Patient characteristics

Our study included 2014 patients (39.11 ± 12.34 years old; males = 1226; females = 788) who underwent FibroTouch assessment between 2015 and 2017. Of these patients, 179 lacked information on γ-GT levels. The baseline characteristics of the 2014 patients are summarized in Table 1. From these patients, 1078, 402, 198, 336 were classified into the F0-F1, F2, F3, and F4 groups, respectively. Significant fibrosis was found in 936/2014 (46.5%) patients, of whom 336 (16.7%) had cirrhosis.

**Table 1.**
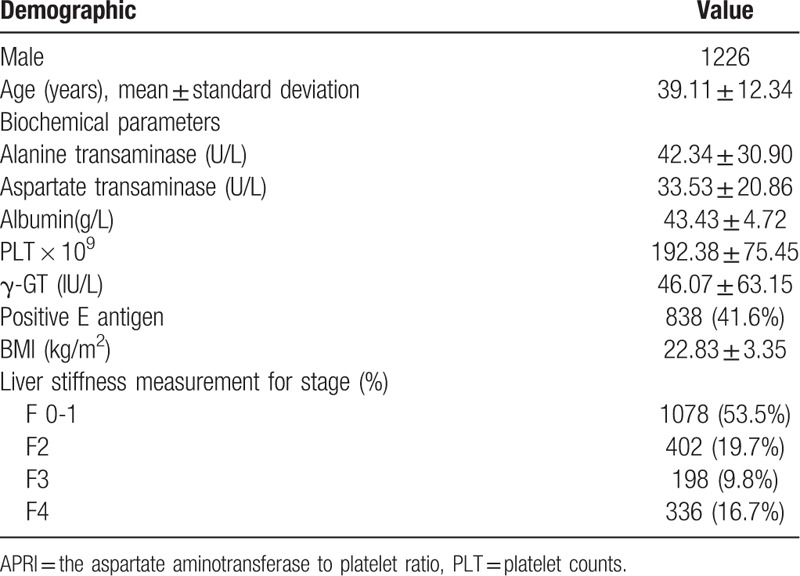


### 3.2 Performance of non-invasive tests for different levels of fibrosis

The three different non-invasive markers of liver fibrosis were compared with TE. The three scores increased progressively with increasing liver stiffness. The analyses are shown in Figure 1 .

**Figure 1:**
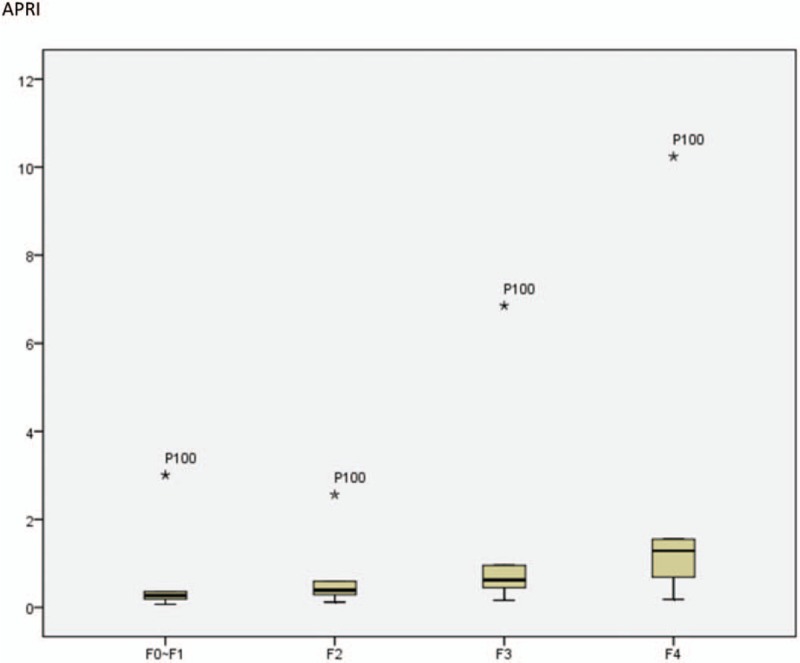
Performance of non-invasive tests for different levels of fibrosis.

**Figure 1 (Continued):**
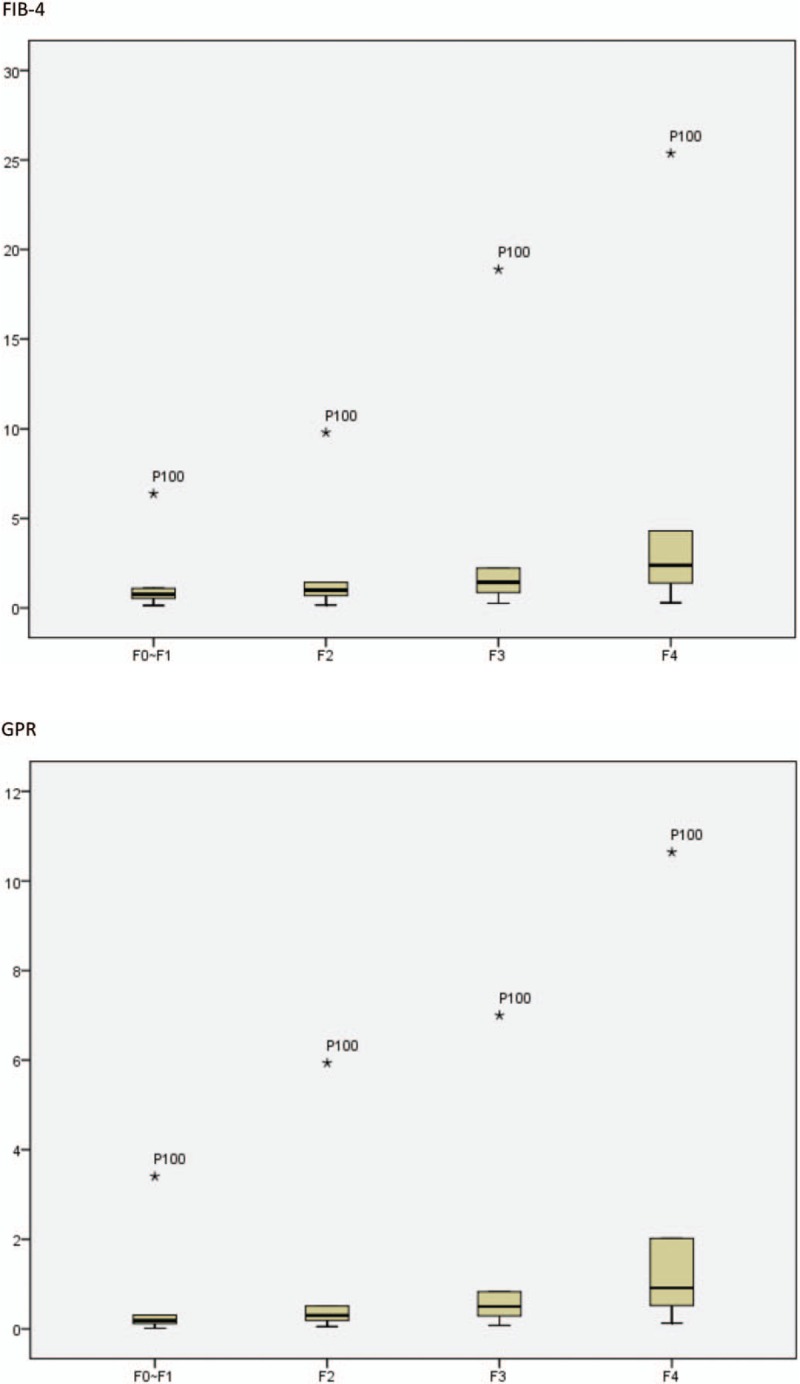
Performance of non-invasive tests for different levels of fibrosis.

### 3.3 AUROC curves for APRI, FIB-4, and GPR in predicting different levels of fibrosis

**Figure 2:**
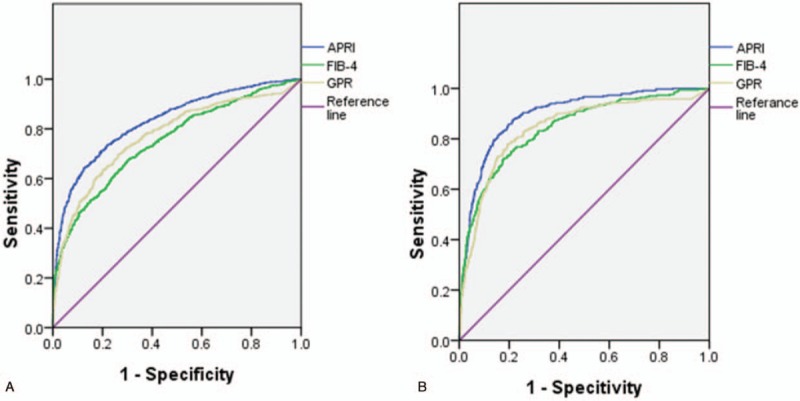
Areas under the receiver operating characteristic curves for aspartate aminotransferase to platelet ratio, fibrosis-4 score, and gammaglutamyl transpeptidase to platelet ratio in predicting different levels of fibrosis.

All non-invasive methods demonstrated high AUROC values for the detection of significant fibrosis and cirrhosis in CHB patients. For the detection of significant fibrosis (FibroTouch > 7.3 kPa), the AUROC curves for APRI, FIB-4, and GPR were 0.83 (95% confidence interval [CI] 0.81–0.85), 0.75 (95% CI 0.73–0.77), and 0.77 (95% CI 0.75–0.79), respectively. For the detection of cirrhosis (FibroTouch > 12.4 kPa), the AUROC curves for APRI, FIB-4, and GPR were 0.90 (95% CI 0.88–0.92), 0.84 (95% CI 0.82–0.87), and 0.84 (95% CI 0.82–0.87), respectively. The AUROC analyses are shown in Figure 2.

### 3.4 Comparison of three non-invasive methods

As seen in Figures 1 and 2 , the APRI score showed the best performance among the three diagnostic methods for the detection of significant fibrosis and cirrhosis. In addition, compared with FIB-4, GPR showed slightly superior performance for the detection of significant fibrosis, though both exhibited the same performance for the detection of cirrhosis. In conclusion, APRI demonstrated excellent ability for predicting significant fibrosis and cirrhosis and may be considered as a good non-invasive alternative, compared to FIB-4 and GPR, for the diagnosis of liver fibrosis and cirrhosis against TE.

### 3.5 APRI cutoff for excluding and predicting fibrosis

**Figure 3:**
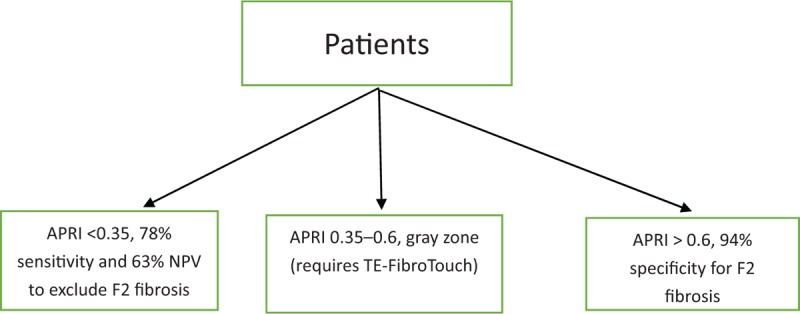
Algorithm of aspartate aminotransferase to platelet ratio for patients.

Discriminant APRI values were determined from the AUROC curves. An APRI cutoff of 0.35 ruled-out significant fibrosis (F ≥ 2) with 78% sensitivity and 63% NPV. Similarly, APRI scores of 0.6, 0.8, and 1.0 detected significant fibrosis (F ≥ 2), bridging fibrosis (F ≥ 3), and cirrhosis (F = 4) with 94%, 95%, and 95% specificity, respectively (Table 2). Thus, based on the results of Table 2, we calculated the cutoff values to exclude and predict significant fibrosis in patients (Fig. 3).

**Table 2:**
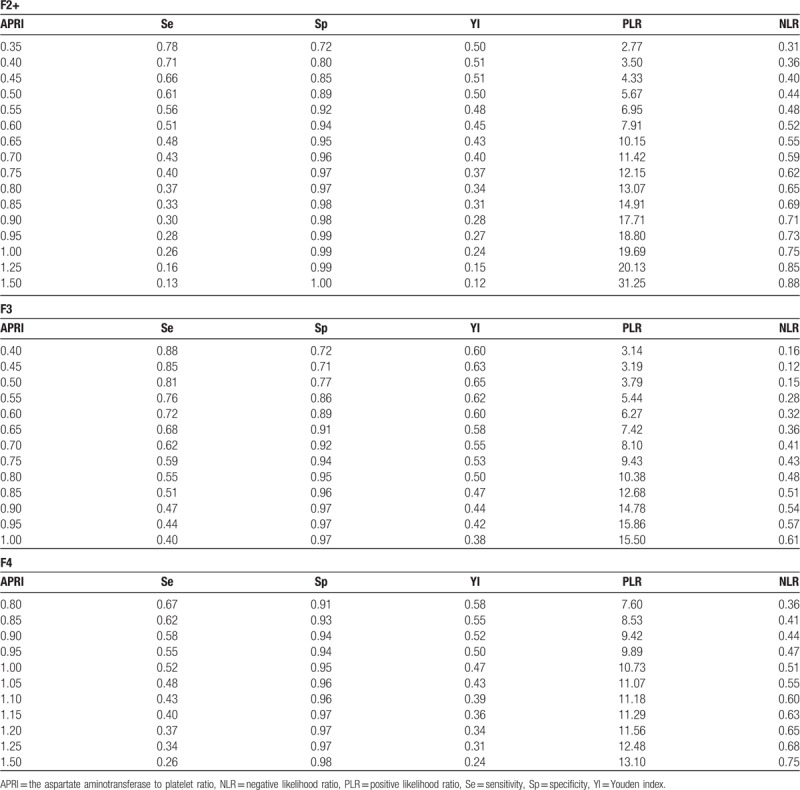
Sensitivity, specificity, Yyouden index, positive likelihood ratio, and negative likelihood ratio of APRI cutoffs for various stages of fibrosis.

## 4 Discussion

Early and accurate assessment of the degree of liver fibrosis is important for the management of patients with CHB,^[13,24–27]^ and crucial for therapeutic decisions and disease prognosis assessment. Given the complications of liver biopsy and cost of TE in resource-limited settings, many studies have evaluated non-invasive tests for liver fibrosis.^[28]^ To the best of our knowledge, however, this study is the first to compare the performances of APRI, FIB-4, and GPR at detecting and diagnosing liver fibrosis against TE in CHB patients in China.

In the present study, APRI, FIB-4, and GPR showed high AUROC values for the detection of significant fibrosis and cirrhosis in CHB patients (0.83 and 0.90, 0.75 and 0.84, and 0.77 and 0.84, respectively). These values were higher than those found in previous research, which reported AUROC values of 0.74 and 0.73 for APRI and 0.78 and 0.82 for FIB-4 for the detection of fibrosis and cirrhosis, respectively.^[29]^ The reason for these differences is likely due to the use of TE as the gold standard rather than liver biopsy.

Figures 2 and 3 indicate that of the 3 diagnostic methods, APRI demonstrated the best performance for the detection of significant fibrosis and cirrhosis. Although GPR showed slightly superior performance to FIB-4 for the detection of significant fibrosis, both showed similar performance for the detection of cirrhosis. In conclusion, APRI exhibited excellent ability to predict significant fibrosis and cirrhosis, and thus may be considered as a suitable non-invasive alternative to FIB-4 and GPR for the diagnosis of liver fibrosis and cirrhosis when used against TE as the gold standard.

The APRI method has also been recently recommended by the WHO for liver fibrosis assessment in CHB, with a cutoff of between 0.5 and 1.5 for significant fibrosis.^[30]^ The use of these WHO threshold values to screen for TE missed many patients with significant fibrosis. In the present study, however, a cutoff of <0.5 ruled out significant fibrosis with a sensitivity of 60% and NLR of 0.44; and a cutoff of ≥1.5 ruled in significant fibrosis with a specificity of 96% and PLR of 3.13. However, only likelihood ratios nearer 10 or 0.1 are regarded as statistically strong for diagnostic evidence.^[31]^ Therefore, for significant fibrosis detection, the above data (NLR and PLR) were too low to provide sufficient statistical support. Thus, we used a lower APRI cut-off score to prevent this problem and avoid the need for TE in CHB patients. Of the 988 patients with an APRI score of <0.35, 777 were assessed as stage normal. Therefore, at a cutoff of 0.35, we could reasonably rule-out significant fibrosis (sensitivity = 78%, NPV = 63%, NLR = 0.31). Similarly, among the 550 patients with an APRI score of >0.6, 478 were assessed at >stage F1, with 94% specificity, 100% PPV, and 7.9 PLR for detecting significant fibrosis (Table 2). Thus, among patients with an APRI value of <0.35 or >0.6, 1255 (82%) were correctly predicted with liver fibrosis. Therefore, we could have avoided TE procedures in patients with a diagnostic accuracy of >80%. However, between 0.35 and 0.6, there was significant variation in the stages of liver fibrosis. Therefore, a score between 0.35 and 0.6 may require TE to accurately stage fibrosis.

Liver fibrosis is a dynamic process that requires serial follow-up in patients. An APRI score is an acceptable, available, and cost-effective method, especially in low-income areas where HBV is prevalent. Imanieh et al demonstrated that APRI value even can evaluate severity of liver fibrosis in children with genetic liver diseases.^[8]^ It is a routine laboratory test that changes with disease progression, thus prompting recalculation of the APRI score and re-staging of the disease by TE or liver biopsy if deemed necessary. This provides economically poor patients and resource-limited governments in rural and remote areas with good diagnosis and follow-up of hepatic fibrosis.

There are several advantages in this study. First, the sample size was reasonably large. Each of the 2014 participants underwent TE and laboratory examinations on the same day. Second, TE was performed by a single skilled investigator and all operators were blind to patient data. Third, elevated serum ALT can influence the accuracy of TE,^[32–34]^ which is significant given that all participants in our study had serum ALT levels at least 3 times greater than the upper limit of normal. Finally, patients treated 6 months prior to this study were excluded, thus avoiding potential interference with TE.^[35]^

This study was somewhat limited by the use of TE rather than liver biopsy. However, current studies have shown good agreement between TE and liver biopsy in patients with CHB. For example, meta-analysis of 2772 CHB patients showed mean AUROC values for TE in the diagnosis of significant fibrosis (F2), severe fibrosis (F3), and cirrhosis (F4) of 0.859, 0.887, and 0.929, respectively, indicating that TE provides good diagnostic accuracy for quantifying liver fibrosis.^[2]^ In conclusion, as a non-invasive method for liver fibrosis diagnosis, APRI may be a reliable predictor of hepatic fibrosis in Chinese patients with CHB compared with TE. We found that an APRI cutoff score of <0.35 and >0.6 was more reliable than the WHO recommended cutoff of <0.5 and >1.5. More importantly, use of an APRI range of <0.35 to >0.6 as a screening tool for significant fibrosis could reduce the need for TE in 61% of patients in Yunnan Province, China. Compared to TE, the cost of APRI is almost 90% cheaper.

## Author contributions

**Conceptualization:** Yan Li.

**Resources:** Jiawei Geng.

**Supervision:** Wei Yue, Ping Wang.

**Writing – original draft:** Li Zhang.

## References

[1] Lu FM Zhuang H Management of hepatitis B in China. Chin Med J 2009;122:1–2. 19187608

[2] Young EC Eun HC Seung UK Performance of transient elastography for the staging of liver fibrosis in patients with chronic hepatitis B: a meta-analysis. Plos One 2012;7:e44930. 23049764 10.1371/journal.pone.0044930 PMC3458028

[3] Deng H Wang C-L Lai J Non-invasive diagnosis of hepatic steatosis using fat attenuation parameter measured by FibroTouch and a new algorithm in CHB patients. Hepat Mon 2016;16:e40263. 27822268 10.5812/hepatmon.40263 PMC5088638

[4] Alizadeh A Mansour-Ghanaei F Roozdar A Laboratory tests, liver vessels color doppler sonography, and FibroScan findings in patients with nonalcoholic fatty liver disease: an observation study. J Clin Imaging Sci 2018;8:12. 29692949 10.4103/jcis.JCIS_93_17 PMC5894278

[5] Huanming X Meijie S Yubao X Comparison of diagnostic accuracy of magnetic resonance elastography and Fibroscan for detecting liver fibrosis in chronic hepatitis B patients: a systematic review and meta-analysis. PLoS One 2017;12:e0186660. 29107943 10.1371/journal.pone.0186660 PMC5673175

[6] Piccinino F Sagnelli E Pasquale G Complications following percutaneous liver biopsy. A multicentre retrospective study on 68,276 biopsies. J Hepatol 1986;2:165–73. 3958472 10.1016/s0168-8278(86)80075-7

[7] van Katwyk S Coyle D Cooper C Transient elastography for the diagnosis of liver fibrosis: a systematic review of economic evaluations. Liver Int 2016;37:851–61. 27699993 10.1111/liv.13260

[8] WHO. Guidelines for the Prevention. Care and Treatment of Persons with Chronic Hepatitis B Infection. Geneva:World Health Organization; 2015. 26225396

[9] Imanieh MH Hakimzadeh M Dehghani SM Aspartate aminotransferase to platelet ratio index and severity of hepatic fibrosis in children. Comp Clin Pathol 2015;24:1611–5.

[10] Kim WR Berg T Asselah T Evaluation of APRI and FIB-4 scoring systems for non-invasive assessment of hepatic fibrosis in chronic hepatitis B patients. J Hepatol 2016;64:773–80. 26626497 10.1016/j.jhep.2015.11.012

[11] Yin Z Zou J Li Q Diagnostic value of FIB-4 for liver fibrosis in patients with hepatitis B: a meta-analysis of diagnostic test. Oncotarget 2017;8:22944–53. 28060754 10.18632/oncotarget.14430 PMC5410276

[12] Elsalam S Habba E Elkhalawany W Correlation of platelets count with endoscopic findings in a cohort of Egyptian patients with liver cirrhosis. Medicine (Baltimore) 2016 Jun;95:e3853. 27281094 10.1097/MD.0000000000003853 PMC4907672

[13] Wai CT Greenson JK Fontana RJ A simple noninvasive index can predict both significant fibrosis and cirrhosis in patients with chronic hepatitis C. Hepatology 2003;38:518–26. 12883497 10.1053/jhep.2003.50346

[14] Sterling RK Lissen E Clumeck N Development of a simple noninvasive index to predict significant fibrosis in patients with HIV/HCV coinfection. Hepatology 2006;43:1317–25. 16729309 10.1002/hep.21178

[15] Lemoine M Shimakawa Y Nayagam S The gamma-glutamyl transpetidase to platelet ratio (GPR) predicts significant liver fibrosis and cirrhosis in patients with chronic HBV infection in West Africa. Gut 2016;65:1369–76. 26109530 10.1136/gutjnl-2015-309260 PMC4975834

[16] Castera L Vergniol J Foucher J Prospective comparison of transient elastography, Fibrotest, APRI, and liver biopsy for the assessment of fibrosis in chronic hepatitis C. Gastroenterology 2005;128:343–50. 15685546 10.1053/j.gastro.2004.11.018

[17] Bousier J Zarski JP de Ledinghen V Determination of reliability criteria for liver stiffness evaluation by transient elastography. Hepatology 2013;57:1182–91. 22899556 10.1002/hep.25993

[18] Castera L Foucher J Bernard PH Pitfalls of liver stiffness measurement: a 5-year prospective study of 13,369 examinations. Hepatology 2010;51:828–35. 20063276 10.1002/hep.23425

[19] Mederacke I Wursthorn K Kirschner J Food intake increases liver stiffness in patients with chronic or resolved hepatitis C virus infection. Liver Int 2009;29:1500–6. 19732330 10.1111/j.1478-3231.2009.02100.x

[20] Myers RP Pomier-Layrargues G Kirsch R Discordance in fibrosis staging between liver biopsy and transient elastography using the FibroScan XL probe. J Hepatol 2012;56:564–70. 22027584 10.1016/j.jhep.2011.10.007

[21] Myers RP Crotty P Pomier-Layrargues G Prevalence, risk factors and causes of discordance in fibrosis staging by transient elastography and liver biopsy. Liver Int 2010;30:1471–80. 20807336 10.1111/j.1478-3231.2010.02331.x

[22] Herrero JI Inarrairaegui M D’Avola D Comparison of the M and XL FibroScan(®) probes to estimate liver stiffness by transient elastography. Gastroenterol Hepatol 2014;37:233–9. 24417906 10.1016/j.gastrohep.2013.10.009

[23] Experts opinions on liver elastic imaging in the diagnosis of liver fibrosis. Chinese Journal of Hepatology 2013;6:420–4.

[24] Castéra L Vergniol J Foucher J Prospective comparison of transient elastography, Fibrotest, APRI, and liver biopsy for the assessment of fibrosis in chronic hepatitis C. Gastroenterology 2005;128:343–50. 15685546 10.1053/j.gastro.2004.11.018

[25] Friedman LS Controversies in liver biopsy: who, where, when, how, why? Curr Gastroenterol Rep 2004;6:30–6. 14720451 10.1007/s11894-004-0023-4

[26] Friedrich-Rust M Ong MF Martens S. Performance of transient elastography for the staging of liver fibrosis: a meta-analysis. Gastroenterology 2008;134:960–74. 18395077 10.1053/j.gastro.2008.01.034

[27] Colloredo G Guido M Sonzogni A Impact of liver biopsy size on histological evaluation of chronic viral hepatitis: the smaller the sample, the milder the disease. J Hepatol 2003;39:239–44. 12873821 10.1016/s0168-8278(03)00191-0

[28] Abdollahi M Pouri A Ghojazadeh M Non-invasive serum fibrosis markers: a study in chronic hepatitis. Bioimpacts 2015;5:17–23. 25901293 10.15171/bi.2015.05 PMC4401163

[29] Xiao G Yang J Yan L Comparison of diagnostic accuracy of aspartate aminotransferase to platelet ratio index and fibrosis-4 index for detecting liver fibrosis in adult patients with chronic hepatitis B virus infection: a systemic review and meta-analysis. Hepatology 2015;61:292–302. 25132233 10.1002/hep.27382

[30] WHO Guidelines Approved by the Guidelines Review Committee Guidelines for the Prevention, and Treatment of Persons with Chronic Hepatitis B Infection. Geneva:World Health Organization. Copyright(c) World Health Organization 2015; 2015.

[31] Chen YP Peng J Hou JL Non-invasive assessment of liver fibrosis in patients with chronic hepatitis B. Hepatol Int 2013;7:356–68. 26201770 10.1007/s12072-013-9439-y

[32] Lucidarme D Foucher J Le Bail B Factors of accuracy of transient elastography (FibroScan) for the diagnosis of liver fibrosis in chronic hepatitis C. Hepatology 2009;49:1083–9. 19140221 10.1002/hep.22748

[33] Myers RP Crotty P Pomier-Layrargues G Prevalence, risk factors and causes of discordancein fibrosis staging by transient elastography and liver biopsy. Liver Int 2010;30:1471–80. 20807336 10.1111/j.1478-3231.2010.02331.x

[34] Kim SU Han KH Park JY Liver stiffness measurementusing FibroScan is influenced by serum total bilirubin in acutehepatitis. Liver Int 2009;29:810–5. 19018979 10.1111/j.1478-3231.2008.01894.x

[35] Vergniol J Foucher J Castera L Changes of non-invasive markers and FibroScan values during HCV treatment. J Viral Hepat 2009;16:132–40. 19175875 10.1111/j.1365-2893.2008.01055.x

